# Number of medically prescribed pharmaceutical agents as predictor of mortality risk: a longitudinal, time-variable analysis in the EPIC-Heidelberg cohort

**DOI:** 10.1038/s41598-023-50487-5

**Published:** 2024-01-02

**Authors:** Verena A. Katzke, Rashmita Bajracharya, Mohamad I. Nasser, Ben Schöttker, Rudolf Kaaks

**Affiliations:** 1 Division of Cancer Epidemiology, German Cancer Research Center (DKFZ), Im Neuenheimer Feld 581, 69120 Heidelberg, Germany; 2 Department of Endocrinology and Metabolism, Molecular Endocrinology Laboratory (KMEB), Odense University Hospital, Odense, Denmark; 3 Department of Clinical Research, University of Southern Denmark, Odense, Denmark; 4 Division of Clinical Epidemiology and Aging Research, German Cancer Research Center (DKFZ), Heidelberg, Germany

**Keywords:** Health care, Risk factors

## Abstract

The number of prescribed medications might be used as proxy indicator for general health status, in models to predict mortality risk. To estimate the time-varying association between active pharmaceutical ingredient (API) count and all-cause mortality, we analyzed data from a population cohort in Heidelberg (Germany), including 25,546 participants with information on medication use collected at 3-yearly intervals from baseline recruitment (1994–1998) until end of 2014. A total of 4548 deaths were recorded until May 2019. Time-dependent modeling was used to estimate hazard ratios (HR) and their 95% confidence intervals (CI) for all-cause mortality in relation to number of APIs used, within three strata of age (≤ 60, > 60 to  ≤ 70 and > 70 years) and adjusting for lifestyle-related risk factors. For participants reporting commonly used APIs only (i.e., API types accounting for up to 80% of medication time in the population) total API counts showed no association with mortality risk within any age stratum. However, when at least one of the APIs was less common, the total API count showed a strong relationship with all-cause mortality especially up to age ≤ 60, with HR up to 3.70 (95% CI 2.30–5.94) with 5 or 6 medications and 8.19 (5.61–11.97) for 7 or more APIs (versus none). Between > 60 and 70 years of age this risk association was weaker, with HR up to 3.96 (3.14–4.98) for 7 or more APIs, and above 70 years it was weakened further (HR up to 1.54 (1.34–1.79)). Multiple API-use may predict mortality risk in middle-aged and women and men ≤ 70 years, but only if it includes at least one less frequently used API type. With advancing age, and multiple medication becomes increasingly prevalent, the association of API count with risk of death progressively attenuates, suggesting an increasing complexity with age of underlying mortality determinants.

## Introduction

Many populations show a high prevalence of medically prescribed drug use among the elderly due to age-related health conditions and morbidities, often with a high percentage of individuals simultaneously using multiple medications to treat more than one health condition^1–7^. Multiple medication use has been speculated to lead to adverse health effects caused by negative pharmacological (“drug-drug” or “drug-disease”) interactions, overmedication, or suboptimal prescriptions. Related to these concerns, the concept of “polypharmacy” was coined, often defined by an arbitrary threshold of five or more medications used simultaneously by one individual^2,8^.

Epidemiologic studies have shown increased risks of all-cause mortality in association with higher numbers of medications used and polypharmacy^2,6–9^. In these studies, however, it is difficult to ascertain whether mortality excess is caused by over-medication or is mostly due to the morbidity conditions for which medications had been prescribed. Independently of this question, however, the number and types of APIs used may be utilized as instrumental proxy indicators for underlying health conditions, in statistical models to predict an individual’s mortality risk and residual life expectancy^10–13^. Studies exploring such use have mostly focused on relatively short-term associations of mortality risk with polypharmacy and did not investigate individuals’ medication trajectories over more prolonged periods of time^14,15^.

To further explore the potential of individuals’ medication data as a possible proxy indicator for health conditions and related mortality risks, we performed an analysis within the Heidelberg component of the European Prospective Investigation into Cancer and Nutrition (EPIC-Heidelberg cohort). In the EPIC-Heidelberg cohort detailed data on medication use and lifestyle-related risk factors were collected at 3-year intervals between 1994 and 2014. Using a time-dependent modelling approach, we present findings from a life course perspective, by examining the relationship between the number of active pharmaceutical ingredients (API) used and all-cause mortality risk over an approximately 20-year follow-up period.

## Methods

### Study design

The current prospective study is based on the EPIC-Heidelberg cohort, which comprises 25,546 men and women from the general population living in the southern German city of Heidelberg and its surrounding municipality at recruitment. The study design and methods for the EPIC prospective cohort study have been described in detail previously^16^. The recruitment period in EPIC-Heidelberg lasted from June 1994 to October 1998^17^, and included women aged 35–65 years and men between the ages of 40–70 years at the time of first inclusion in the study. Baseline examinations included measurement of anthropometric indices by trained staff, assessment of lifestyle factors and dietary habits via comprehensive questionnaires, and collection of a blood sample. The questionnaire data on non-dietary variables included those on education and socio-economic status, current job, current and past occupation, history of previous illness, lifetime history of tobacco smoking (duration [years], average intensity [cigarettes/day], time since quitting for ex-smokers [years]), lifetime history of consumption of alcohol beverages, medication use and physical activity. Informed consent was obtained from all participants at baseline, the Medical Ethics Committee of the Heidelberg University and the International Agency for Research on Cancer (IARC) approved the EPIC-Heidelberg study. After baseline recruitment, individuals were asked to return up to six additional follow-up questionnaires at approximate 3-year intervals, until end of 2014. Standardized core questions derived from baseline were asked in each follow-up round, including assessment of medication intake, resulting in six follow-up waves with detailed medication data available.

### Medications classification and polypharmacy definition

Information about the use of medications was collected at baseline and then in up to six follow-up questionnaires that were sent to the study participants every 3 years. On every occasion, participants were asked to report all prescribed medications and hormones that they had used or applied regularly over the last four weeks, including those purchased over the counter. Medication names, dosages and types were reported. For medications present on the German market (“Gelbe Liste”: https://www.gelbe-liste.de/atc), the data were coded through the Anatomical Therapeutical Chemical (ATC) classification system^18^. Multiple medication-use in our cohort was defined as the number of different active pharmaceutical ingredients (APIs) used; thus, medications containing multiple APIs were counted as multiple medications, using the coding system developed by Schöttker et al.^19^. We excluded medications that did not require medical prescription—i.e. food supplements, minerals, and vitamins, homeopathic or anthroposophical drugs, some herbal drugs, and non-systematically acting APIs that have no known adverse drug reactions (ADRs) other than local reactions—from the present analyses (Appendix 1). Furthermore, we also excluded sex hormones and modulators of the genital system (GO3), i.e. hormone replacement therapy, oral contraceptives, as generally these are prescribed for contraception or for menopausal symptom relief but not, in the vast majority of cases, for the primary purpose of treating a morbidity condition.

### Outcome classification

Participants in EPIC-Heidelberg were followed from study entry (1994–1998) until date of death, loss to follow-up or censoring date (31/05/2019), whichever occurred first. Mortality outcomes were ascertained through regular record linkages with municipal registries, which provide information about vital status, as and with regional health offices for more detailed death certificates.

### Statistical analysis

Our analyses were conducted using an extension of Cox proportional hazard model, namely a time-dependent Cox model, to estimate the effect of time-varying covariate on survival^20^ . Briefly, our API count was a longitudinal, time-varying variable based on the multiple follow-up questionnaires. Entry times were left-truncated at age-at-recruitment. In all models, the individuals’ age was used as the underlying time scale.

The statistical analyses were restricted to APIs classified at level 2 of ATC coding (e.g., “A10”, for all glucose-lowering medications). The total number of APIs used by each individual was updated at each of the six follow-up rounds after baseline. We categorized an individual as being in the polypharmacy group if he or she was taking 5 or more API in order to compare our results to previous investigations. To determine which API individuals had been taking most frequently and for the longest duration we tabulated the total person-years spent on API across age strata (age windows of ≤ 60, 60- ≤ 70, and > 70 years). Out of 25,520 individuals, 19,677 (77% of the cohort) had complete medication information at baseline and across all the six follow-up questionnaires (FUP1, FUP2, …., FUP6), 12% missed one, 6% missed two and 5% had missing data on medication use on three or more follow-up occasions. The numbers of missing values were equally distributed across the three age windows. In our statistical modeling, from baseline till FUP6, the last observation on medication use was carried forward up until either the subsequent follow-up ascertainment of medical use or, if a participant did not respond to a follow-up questionnaire, for 3 further years (or date of death, whatever came first). For all participants who had completed the 6th follow-up questionnaire (FUP6), this last observation was carried forward until the final censoring date of May 2019 (or date of death, whichever came first). The median duration between FUP6 and date of censoring, or death, was 5.5 years (inter-quartile range, 5.3–5.7 years).

All proportional hazards analyses were adjusted for age, which is implicit in our left-truncated age-as-time-scale model. In addition, models were adjusted for several baseline-covariates, including body mass index (BMI), physical activity, smoking intensity and history [lifetime duration of smoking (years), average daily cigarettes, and time since quitting (in years) for former smokers], and highest level of formal education as a proxy for socioeconomic status. We used Schoenfeld residuals and the “cox.zph” function of the “survival” package in R-studio to determine possible violations of the proportional hazards assumption for each variable. The proportional hazards assumption appeared violated for mortality risk in relation to the number of APIs, indicating that the relative risk association of API count with mortality was not constant with age; we therefore allowed the hazard ratio for API to change with age, and estimated separate hazard ratios for each of three age windows: below age 60, between 60 and 70 years, and 70 years and above. We also tested for interactions between API count and sex regarding mortality risks, but we found no evidence for such interaction.

Since the concept of multiple medication use such as the polypharmacy score or other, combines multiple medications of differing clinical effect, we investigated which medications were responsible for the association of multiple medication-use with mortality, by building a similar score using only the APIs responsible for 80% of the person-years of API use by the participants (further referred to as “common” APIs), and comparing the effect of this score to one constructed using the remaining 20% of API use, further referred to as “uncommon” APIs, Appendix 2.

All statistical analyses were carried out using R (version 4.0.1, 2020)^21^.

### Ethics declaration

This project is covered by the ethical approval for the EPIC-Heidelberg cohort (Ethical Committee of the Medical Faculty Heidelberg, reference number 13/94).

### Consent for participation

Informed consent was obtained from all participants at baseline (1994–1998).

## Results

Baseline characteristics of the study population (N = 25,540) are displayed in Table 1. A total of 4,548 deaths (18% or study participants) were recorded, over an average of about 20 years of prospective follow-up. The mean ages at recruitment were 50 years (SD = 8 years, interquartile range (IQR) = 43–56 years) and 56 years (SD = 7 years, IQR = 53–62 years), and the mean follow-up durations were 22 years (SD = 2 years, IQR = 21–23 years) and 14 years (SD = 6 years, 10–19 years), respectively, for the participants alive at the end of the study and for those deceased. Although men represented only 43% of the study participants, the proportion of men in the deceased group was 64%. Furthermore, thepercentage of current smokers (32%) was higher in the deceased group than in the alive group (21%). The prevalence of polypharmacy was more than three times higher in the deceased group (12%) compared to the alive group (4%), and the prevalence for self-reported diseases at baseline recruitment was also higher in the deceased group.

**Table 1:** Baseline characteristics of the EPIC-Heidelberg cohort (N = 25,540).

	Participants alive	Participants deceased
	n (%)/mean (SD)	n (%)/mean (SD)
N	20,992 (82.1%)	4548 (17.8%)
Age (years) at recruitment	49.7 (7.8)	56.5 (6.7)
Mean age of censoring	71.8 (8.1)	70.9 (9.3)
Duration of follow-up (years)	22.1 (1.9)	14.4 (6.1)
Sex (males)	9045 (43.1%)	2288 (63.3%)
BMI (kg/m^2^)	25.8 (4.1)	27.5 (4.6)
Smoking		
Never	9098 (43.3%)	1562 (34.3%)
Former	7362 (35.1%)	1506 (33.1%)
Smoker	4472 (21.3%)	1465 (32.2%)
Unknown	60 (0.2%)	15 (0.3%)
Highest school level		
None	105 (0.5%)	48 (1.1%)
Primary school completed	5437 (25.9%)	1876 (41.2%)
Technical/professional school	7197 (34.3%)	1458 (32.1%)
Secondary school	1569 (7.4%)	241 (5.3%)
Longer education (incl. university deg.)	6664 (31.7%)	922 (20.2%)
Physical activity		
Inactive	4968 (23.6%)	755 (16.6%)
Moderately inactive	5823 (27.7%)	1411 (31.0%)
Moderately active	8261 (39.3%)	2062 (45.3%)
Active	1940 (9.2%)	320 (7.0%)
Alcohol intake (mg/day)	16.6 (20.7)	23.1 (32.4)
API count*		
0	11,402 (54.3%)	1739 (38.2%)
1—4	8855 (42.1%)	2264 (49.7%)
Polypharmacy (≥ 5)	735 (3.5%)	545 (11.9%)

*Number of active pharmaceutical ingredients taken.

**Figure 1:**
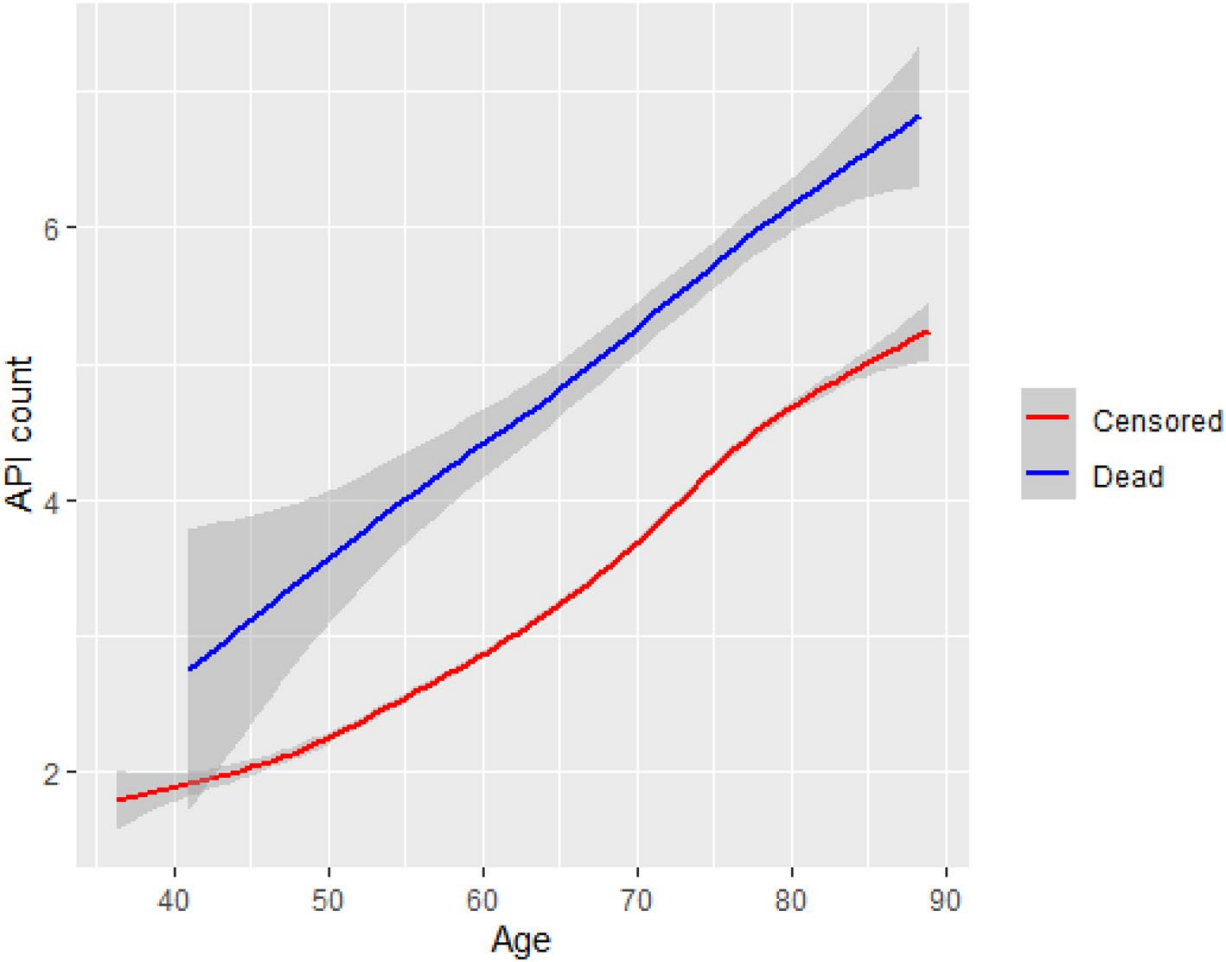
API trajectories by outcome in the longitudinal EPIC-Heidelberg cohort. Smoothed trajectory ‘spaghetti’ plot, using locally estimated scatterplot smoothing (LOESS), excluding G03.

After excluding four ATC code groups (Appendix 1), 80 percent of medication use time was related to only a relatively small subset of medication classes (Appendix 2A; two-level ATC categories), which included only 16 basic ATC medication classes up to the age of 60, and only 12 basic ATC medication groups when EPIC study participants were older than 70. The most common prescriptions were for medications targeting the cardiovascular system (C-category), which represented 33.4% of medication use up to age 60, increasing to 47.4% when participants were older than 70. The use of anti-thrombotic medications (B01) gradually rose from 4.8% of time below age 60 to 11.9% after age 70, whereas the relative part of medications used for thyroid diseases diminished from 11.1% to 4.6%. Other categories of commonly used APIs included glucose-lowering medications (A10, 2.4–3.9% depending on age window), medications used to treat gastro-intestinal reflux problems (A02, 3.7–4.3%) and APIs with anti-inflammatory and/or anti-rheumatic actions (M01, 4.7–2.7%). The group of less commonly used medications, which jointly accounted for the remaining 20% of API use, included a total of 29 API classes described in Appendix 2B, where each individual class accounted for only 0.3% to 2.5% of estimated time of use (Appendix 2A). The numbers of APIs used simultaneously, increased with age, almost linearly (Fig. 1).

Models adjusted for age, BMI, physical activity, smoking history, highest school level as a proxy for socioeconomic status, and self-reported myocardial infraction, diabetes, stroke, and hypertension at baseline recruitment showed significant associations between increasing API count and mortality rates (Table 2). Yet, the strength of association between API count and risk of death varied across different age windows. Below the age of 60, we observed a hazard ratio (HR) of 1.87 (95% CI: 1.20, 2.91) for those using 4 APIs, 2.50 (1.67–3.73) for those using 5 or 6 APIs and 6.28 (95% CI: 4.37, 9.03) for those using 7 or more APIs, relative to individuals who did not use any medications. For the age window of 60–70 years the HR estimates were moderately lower. For 70 years and above, the number of APIs used overall had no major association with mortality risk, except for individuals using seven or more APIs [HR = 1.35 (95% CI: 1.17, 1.55)].

**Table 2:** Hazard ratios (and 95% CI) for API count and mortality across categories, for individuals using all API, only common API, or at least one uncommon API.

API count categories	Model	Reference*	1	2	3	4	5 or 6	7 or more	Polypharmacy (≥ 5)
Age ≤ 60									
All API	Crude	1.00	1.04 (0.71–1.47)	1.15 (0.79–1.67)	1.48 (0.98–2.24)	2.00 (1.29–3.09)	2.76 (1.87–4.07)	7.46 (5.29–10.5)	4.32 (3.23–5.78)
	Full	1.00	1.05 (0.74–1.49)	1.13 (0.78–1.64)	1.40 (0.92–2.12)	1.87 (1.20–2.91)	2.50 (1.67–3.73)	6.28 (4.37–9.03)	3.74 (2.75–5.08)
Using only common API	Crude	1.00	0.72 (0.46–1.11)	0.91 (0.57–1.45)	0.85 (0.44–1.64)	1.25 (0.61–2.59)	1.32 (0.57–3.04)	2.61 (0.83–8.16)	1.81 (1.08–3.04)
	Full	1.00	0.87 (0.58–1.30)	0.87 (0.56–1.37)	1.07 (0.63–1.81)	0.95 (0.48–1.90)	1.39 (0.76–2.56)	1.65 (0.67–4.07)	1.45 (0.86–2.45)
Using at least one uncommon	Crude	1.00	1.88 (1.06–3.34)	2.04 (1.16–3.57)	2.11 (1.16–3.85)	3.41 (2.01–5.76)	3.99 (2.52–6.34)	9.40 (6.57–13.45)	6.39 (4.68–8.73)
	Full	1.00	2.02 (1.14–3.58)	2.09 (1.19–3.67)	2.08 (1.14–3.80)	3.36 (1.97–5.74)	3.70 (2.30–5.94)	8.19 (5.61–11.97)	5.67 (1.67–3.21)
	PY	108,404	36,113	29,516	16,260	9907	9111	4546	13,657
	deaths	125	44	38	29	25	34	47	81
Age > 60 and ≤ 70									
All API	Crude	1.00	1.03 (0.80–1.32)	0.92 (0.71–1.20)	1.18 (0.91–1.53)	1.38 (1.05–1.81)	1.75 (1.39–2.21)	4.02 (3.28–4.92)	2.68 (2.23–3.22)
	Full	1.00	1.06 (0.82–1.36)	0.93 (0.71–1.22)	1.18 (0.90–1.53)	1.30 (0.99–1.70)	1.57 (1.24–1.99)	3.29 (2.65–4.08)	2.28 (1.89–2.76)
Using only common API	Crude	1.00	0.95 (0.72–1.25)	0.69 (0.49–0.97)	0.90 (0.64–1.27)	1.29 (0.91–1.84)	1.17 (0.81–1.70)	2.19 (1.40–3.40)	1.48 (1.12–1.97)
	Full	1.00	1.03 (0.79–1.35)	0.63 (0.45–0.89)	0.92 (0.66–1.28)	1.07 (0.76–1.52)	1.03 (0.73–1.46)	1.61 (1.06–2.44)	1.19 (0.90–1.58)
Using at least one uncommon	Crude	1.00	1.27 (0.77–2.09)	1.93 (1.36–2.75)	1.70 (1.20–2.43)	1.63 (1.14–2.35)	2.21 (1.68–2.89)	4.63 (3.75–5.73)	3.42 (2.82–4.15)
	Full	1.00	1.28 (0.77–2.11)	1.96 (1.38–2.79)	1.71 (1.21–2.44)	1.58 (1.10–2.28)	2.06 (1.57–2.71)	3.96 (3.14–4.98)	3.02 (2.46–3.70)
	PY	52,999	24,808	23,287	18,596	14,077	16,613	11,326	27,939
	deaths	197	92	77	81	73	114	184	298
Age > 70									
All API	Crude	1.00	0.58 (0.46–0.72)	0.48 (0.38–0.61)	0.61 (0.50–0.75)	0.71 (0.58–0.86)	0.96 (0.82–1.12)	1.59 (1.39–1.81)	1.28 (1.13–1.44)
	Full	1.00	0.64 (0.51–0.80)	0.53 (0.42–0.66)	0.65 (0.53–0.80)	0.72 (0.59–0.88)	0.92 (0.79–1.07)	1.35 (1.17–1.55)	1.14 (1.01–1.29)
Using only common API	Crude	1.00	0.53 (0.42–0.68)	0.43 (0.33–0.57)	0.54 (0.42–0.70)	0.53 (0.41–0.70)	0.76 (0.62–0.94)	0.92 (0.71–1.19)	0.81 (0.68–0.97)
	Full	1.00	0.62 (0.49–0.79)	0.50 (0.38–0.65)	0.60 (0.46–0.77)	0.54 (0.42–0.71)	0.71 (0.58–0.88)	0.77 (0.59–1.01)	0.73 (0.61–0.88)
Using at least one uncommon	Crude	1.00	0.92 (0.58–1.45)	0.66 (0.45–0.96)	0.76 (0.56–1.03)	1.02 (0.79–1.31)	1.14 (0.95–1.36)	1.79 (1.56–2.05)	1.53 (1.35–1.74)
	Full	1.00	0.98 (0.62–1.56)	0.67 (0.46–0.99)	0.79 (0.58–1.07)	1.02 (0.80–1.31)	1.10 (0.92–1.32)	1.54 (1.34–1.79)	1.37 (1.20–1.57)
	PY	23,065	10,147	10,744	11,021	10,196	15,783	15,502	31,285
	deaths	421	98	89	119	135	299	516	815

Crude model—adjusted for age and sex.

Full model—adjusted for age, sex, BMI, smoking history (average daily cigarettes, duration, and time since quitting), physical activity, highest level of schooling.

Death count is the number of individual deaths while in the specific API category. Individuals can move across API categories throughout follow-up.

^a^Active pharmaceutical ingredient.

^b^Reference category is no API intake.

**Figure 2:**
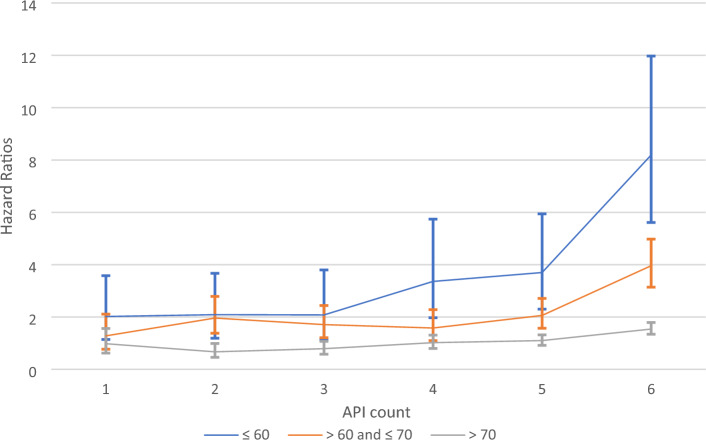
Hazard ratios (with 95% confidence intervals) for API count and mortality across age categories, for individuals using at least one uncommon API compared to no API intake.

Comparing commonly used to less used medications, we observed that the relative mortality hazards were increased especially for those individuals who used at least one uncommon API type (Table 2) (Fig. 2). For individuals aged 60 years or younger, the HR for five or six APIs versus none is 1.39 (95% CI: 0.76, 2.56), when no uncommon APIS are present, and 1.65 (95% CI: 0.67–4.07) for those using 7 or more APIs. However, when at least one of the APIs was of a less commonly used type the HRs increased to 3.70 (95% CI: 2.30, 5.94) and 8.19 (95% CI: 5.61–11.97), respectively. Also, in the age window of > 60 to ≤ 70 years an increasing API count was related to increasing mortality risk only when medication use included at least one less common API type, but with more moderate HRs as compared to the younger age window. Above age 70, even when at least one less common API type was used, higher API counts were not related to any major increase in mortality risk unless 7 or more APIs were used (HR = 1.54; 95%CI: 1.34–1.79).

## Discussion

Using a time-dependent modelling approach we assessed the relationships between age-varying medication use and all-cause mortality in a general population cohort of men and women initially 35 to 65 years of age (40–65 years for men). Adjusting for age, smoking history, BMI and level of formal education we found that mortality risk increased significantly with increasing number of APIs, relative to no medication use, but only if at least one of the APIs belonged to medication classes that were used less commonly. Furthermore, higher numbers of APIs were related to the most prominent increase in mortality risk when the study participants were below the age of 60 and to a smaller increase when they were between ages of 60 and 70 years, whereas above the age of 70 the number of APIs no longer showed a clear association with mortality risk. Overall, our data indicate that API count may be a useful predictor for mortality risk, especially below the age of 70 years.

Reviews and meta-analyses of epidemiologic studies showed an average relative mortality risk of about 1.3 for individuals falling into a “polypharmacy” category, mostly defined by use of ≥ 5 medications, relative to individuals using a smaller number of medications^6,8,9^. Based on a smaller subset of studies, meta-analyses further showed an all-cause mortality HR of 1.08 per single additional medication used, and showed a pooled adjusted risk ratio of 1.24 (95% CI 1.10–1.39) for studies using a threshold of 1–4 medications, 1.31 (1.17–1.47) for 5 medications, 1.59 (1.36–1.87) for 6–9 medications, and 1.96 (1.42–2.71) for 10 or more medications^6,7^. The studies included in these meta-analyses reported associations of variable strength between number of medications used, polypharmacy and mortality risk, and were also highly diverse with regard to overall study size, types of study population (e.g., community-dwelling individuals within variable age ranges; people living in care institutions; geriatric patients; hospital discharge patients), and different definitions for individuals classified as being exposed to polypharmacy or to a comparison (reference) group. In addition, studies varied widely regarding adjustments made for confounding by indication or for confounding by general health-related risk factors: some of studies included adjustments for selected comorbidities or for more comprehensive multi-morbidity scales such as Charlson’s Comorbidity Index. Other studies used more novel methods such as propensity score matching to assess whether polypharmacy itself had causal effects on mortality risk independently of the underlying health conditions. The heterogeneity in the analytical approaches used may reflect differences in the original study objectives, either aiming to examine medication use as a potential cause of death, or rather focusing on medications prescribed as a substitute indicator for health conditions in the prediction of mortality risk. In our analyses, we chose to calculate HRs without any adjustment for self-reported co-morbidities. We believe that medication data may be used merely as an instrument to predict mortality risk and life expectancy, as a substitute indicator for underlying morbidities that are the more likely risk determinants.

A general issue with the studies modeling adverse health effects of polypharmacy as a monotonous function of a mere count of different APIs used, is the implicit assumption that each additional API, irrespective of its type, would increase the risk of the adverse effect to an equal extent. Many studies may have used this assumption to allow any form of statistical modeling at all, as often these studies were too small to allow modeling in relation to more specific categories of medication exposures. However, our observation that mortality risk was associated more strongly with APIs that were used less commonly, as compared to commonly used APIs, indicates that this assumption represents an over-simplification that may lead to suboptimal modeling of adverse health effects. Recent analyses within very large-scale, national drug prescription databases in Sweden have provided further confirmation for this, showing relatively strong risk associations when focusing on more specific medication types at the subgroup (3rd or 4th) coding level of the ATC classification, but less clear-cut associations for other medications within a same class at the more aggregated, 2nd ATC coding level^11,12^. In these Swedish studies, very strong associations with mortality risk were noted, for example, for serotonin antagonists (N06AB) and for propulsive drugs (A03F), frequently prescribed for nausea and vomiting and likely indicators for malignancy and associated treatment, or sub-types of diuretics (C03CA, C03DA) prescribed for severe conditions such as heart and kidney failure, whereas many other drugs in the “N”, “A” or “C” categories of the ATC system showed no significant association with risk. These examples underlined the importance of information carried in the 3rd and 4th levels of the ATC code (specifically: the pharmacological subgroup, over the information carried by the main anatomical group) when using medication data for the purposes of predicting mortality risk^11,12^.

Unfortunately, in spite of including a fairly large number of (> 4500) mortality events out study was far too small to allow analyses on mortality risk in relation to a more detailed and more specific classification of APIs on the 3rd or 4th level of the ATC classification, as was done in the Swedish national database studies. Nonetheless, our findings do show that, to a large extent, associations between medication use and increased risk of death may be driven by less commonly used drugs that may to indicate more serious underlying medical conditions. Interestingly, however, our data also indicate that especially for individuals using at least one less common medication type, risk of death is also associated with total API count. The latter suggests that also the more common medication types (e.g. analeptics, psycholeptics) may gain predictive power for mortality risk for individuals treated for a more serious morbidity condition.

Compared to previous studies, a unique feature of our investigation is that it was based on regular, prospective data collections over a 20-year period, for a community-dwelling population of women and men who were initially between 35 and 65 years of age, applying a time-dependent modelling approach to examine the prospective relationships between (changes in) medication use and mortality risk. This longitudinal approach, combined with fairly large number of mortality outcomes over time, allowed us to examine risk associations within different age windows, as participants in the EPIC-Heidelberg grew older. Medication use was defined by a numerical threshold depending on the number of APIs contained in each medication, as a more precise definition^19^. Our data also allowed careful adjustments for possible confounding by relevant lifestyle factors. An interesting finding from our analyses is that with advancing age the association of mortality risk with number of APIs used progressively appeared to flatten, a phenomenon that, to our knowledge, has not been given much attention in previous studies on multiple medication use, polypharmacy scores and mortality risk. While polypharmacy, defined as the use of 5 or more APIs, reached a high prevalence above the age of 70 years, we also observed that 80% of medications within this age group were concentrated in only 12 frequently used API classes. Most of these medications were for APIs used as preventive medications, e.g. to avoid worsening of highly prevalent conditions related to the cardiovascular system (e.g. hypertension) and blood coagulation, or used as pain killers to combat symptoms related to muscular-skeletal problems, which are mostly low-risk conditions. Other studies have also shown that among elderly individuals with high prevalence of medication use, most medications belonged to a very restricted number of ATC classes^22–26^. Furthermore, it has been reported that among elderly individuals (residents of nursing facilities) the association of polypharmacy with mortality risk may be modified by other health conditions such as frailty, and that risk of death was increased mostly by frailty and disability, whereas (hyper-)polypharmacy was associated with higher risk of death only among non-frail study participants^27^.

A minor limitation of our investigation is the proportion of missing medication information across follow-ups, which adds up to 23% of at least one time missing medication use, although only a small proportion of study participants had missing information on three or more follow-up occasions (5%). Theoretically, a selective drop-out of study participants could have biased our hazard ratio estimates for mortality, if absence of response to a follow-up questionnaire was non-random and in itself informative about an individual’s health status (“informative missings”). However, information from follow-up questionnaires was carried forward only for a limited amount of time, which very much should mitigate any risk for such bias. Also, to further explore informative missingness as a potential source of bias, we additionally applied more complex approaches for the joint modelling of medication exposures and mortality risk^28^. The results from this more complex modelling (results not shown) were practically identical to those from the simpler time-variable Cox modeling presented in the present manuscript.

In summary, our longitudinal analyses show a complex quantitative relationship between multiple medication-use and risk of all-cause mortality in a general population cohort of middle-aged and older individuals. Our data indicate that classifying individuals into risk categories based on the mere number of medications used, as with the “polypharmacy” concept, represents an over-simplification that may lead to sub-optimal modeling of adverse health effects. More accurate risk modeling would require accounting for a higher level of detail and specificity for medication types described for severe *vs.* less severe health conditions. Furthermore our data show that with advancing age, as multiple medication use becomes increasingly prevalent, the prediction of mortality risk becomes increasingly complex and may be less directly related to a mere number of APIs used. This phenomenon, and the specific relationships of mortality with low- *vs*. high-risk medication types, should be explored further in context of very large-scale studies, e.g. using national drug prescription databases^11–13,23–26^.

### Supplementary Information

Supplementary Information.

## Data Availability

EPIC-Heidelberg was launched in the 1990s. Unlike in new studies that we run today, public access to data from the EPIC population was not part of the study protocol at that time. Thus, the data protection statement and informed consent of the EPIC participants do not cover the provision of data in public repositories. Nevertheless, upon special request data may be made available for (a) statistical validation by reviewers and (b) pooling projects under clearly defined and secure conditions and based on valid data transfer agreements.

## Abbreviations

API: Active pharmaceutical ingredient
ATC: Anatomical therapeutic chemical
ADR: Adverse drug reaction
BMI: Body mass index
CI: Confidence interval
EPIC: European Prospective Investigation into Cancer and Nutrition
HR: Hazard ratio
ICD: International Classification of Diseases
MI: Myocardial infarction
