# Supplementary material for: Number of medically prescribed pharmaceutical agents as predictor of mortality risk: a longitudinal, time-variable analysis in the EPIC-Heidelberg cohort
Source: Sci Rep. 2024 Jan 2;14:106. doi: 10.1038/s41598-023-50487-5 (PMC10762119; doi:10.1038/s41598-023-50487-5)
Supplement: Supplementary file 1 — Supplementary Information. [file 41598_2023_50487_MOESM1_ESM.docx]

# **Legends**

***Appendix 1****: ATC codes or ATC code groups excluded from the count of drugs*

***Appendix 2A****: Drugs by person-years in three age categories across baseline and six follow-up rounds, representing up to 80% of person-years (A) and 20 most prevalent uncommon drugs (B)*

***Appendix 2B:*** *Drug classification corresponding to ATC code*

***Appendix 3:*** *Most commonly prescribed drugs (ATC Classification) in the EPIC-Heidelberg cohort, by follow-up and sex, including G03*

Pn = participating number of subjects, FUP = follow-up

***Appendix 1:*** *ATC codes or ATC code groups excluded from the count of drugs*

| Exclusion Criteria | ATC code |
| --- | --- |
| Food supplements | A11, A12, V06, A13, A02X, C10AX06 |
| Homeopathic or anthroposophical drugs | V60, N05H, A01AH, A03AH, A04AH, A05AH, A05BH, A07XH, A08AH, A09AH, A10XH, B03XH, C01AH, C01CH, C01EH, C02KH, C03XH, C04AH, C05AH, C05CH, C06AH, D05BH, D10BH, G02CH, G04BH, G04CH, H03BH, H05AH, L01CH, L03AH, M02AH, M04AH, M09AH, N01BH, N02BH, N02CH, N04AH, N06AH, N07CH, N07XH, R01AH, R01BH, R03DH, R05CH, R05FH, R05XH, R07AH, S02DH, R05CP, R05DP02, R05DP04, R05DP07 |
| Herbal drugs without systemic action | A03AP, C05AP, R05DP |
| Non-systemic drugs | D02, D03, D04, D08, D09, D11, R02, R04, S01, V04, V07, V08, V09, V20, D01A, D06A, M02B, P03A, P03B, A01AA, A01AP, D07AA, D07BA, D07CA, D07XA, G02CD, M09AX, S02DC, C10AX13 |

***Appendix 2A:*** *Drugs by person-years in three age categories across baseline and six follow-up rounds, representing up to 80% of person-years (A) and 20 most prevalent uncommon drugs (B)*

| (A) Common drugs | | | |  | | | |  | | | |
| --- | --- | --- | --- | --- | --- | --- | --- | --- | --- | --- | --- |
| ≤ 60 years | | | | 60 – 70 years | | | | > 70 years | | | |
| Drug | Person years | Person years % | Cum Person years % | Drug | Person years | Person years % | Cum Person years % | Drug | Person years | Person years % | Cum Person years % |
| C09 | 21758 | 11,4 | 11 | C09 | 43807 | 15,2 | 15 | C09 | 42502 | 15,9 | 16 |
| H03 | 21077 | 11,1 | 22 | C07 | 34528 | 12,0 | 27 | C07 | 31789 | 11,9 | 28 |
| C07 | 20745 | 10,9 | 33 | C10 | 26077 | 9,1 | 36 | B01 | 31701 | 11,9 | 40 |
| C10 | 11085 | 5,8 | 39 | B01 | 24917 | 8,7 | 45 | C10 | 26147 | 9,8 | 49 |
| N02 | 10165 | 5,3 | 45 | H03 | 18560 | 6,4 | 51 | C08 | 13993 | 5,2 | 55 |
| N06 | 9758 | 5,1 | 50 | C08 | 13345 | 4,6 | 56 | H03 | 12314 | 4,6 | 59 |
| B01 | 9084 | 4,8 | 54 | A02 | 10967 | 3,8 | 60 | C03 | 11979 | 4,5 | 64 |
| M01 | 8941 | 4,7 | 59 | N06 | 10911 | 3,8 | 64 | A02 | 11391 | 4,3 | 68 |
| A02 | 7120 | 3,7 | 63 | C03 | 10507 | 3,6 | 67 | A10 | 10455 | 3,9 | 72 |
| G02 | 6519 | 3,4 | 66 | A10 | 10280 | 3,6 | 71 | G04 | 8461 | 3,2 | 75 |
| C08 | 5406 | 2,8 | 69 | N02 | 9305 | 3,2 | 74 | N06 | 8365 | 3,1 | 78 |
| R03 | 4701 | 2,5 | 72 | M01 | 9088 | 3,2 | 77 | M01 | 7138 | 2,7 | 81 |
| C03 | 4681 | 2,5 | 74 | G04 | 8419 | 2,9 | 80 |  |  |  |  |
| A10 | 4656 | 2,4 | 76 |  |  |  |  |  |  |  |  |
| N05 | 3977 | 2,1 | 79 |  |  |  |  |  |  |  |  |
| M04 | 3557 | 1,9 | 80 |  |  |  |  |  |  |  |  |

| (B) Uncommon drugs | | | |  | | | |  | | | |
| --- | --- | --- | --- | --- | --- | --- | --- | --- | --- | --- | --- |
| G04 | 3383 | 1,8 | 82 | M04 | 7296 | 2,5 | 83 | M04 | 6756 | 2,5 | 83 |
| R06 | 2835 | 1,5 | 84 | C01 | 5341 | 1,9 | 84 | C01 | 5250 | 2,0 | 85 |
| C01 | 2340 | 1,2 | 85 | R03 | 5172 | 1,8 | 86 | N02 | 5082 | 1,9 | 87 |
| J01 | 2328 | 1,2 | 86 | N05 | 3870 | 1,3 | 88 | R03 | 3944 | 1,5 | 89 |
| R05 | 2310 | 1,21 | 87 | C02 | 3587 | 1,2 | 89 | C02 | 3350 | 1,3 | 90 |
| A05 | 1918 | 1,01 | 88 | H02 | 2467 | 0,9 | 90 | N05 | 3032 | 1,1 | 91 |
| H02 | 1887 | 0,99 | 89 | A05 | 2158 | 0,7 | 90 | H02 | 2610 | 1,0 | 92 |
| C02 | 1817 | 0,95 | 90 | N03 | 1875 | 0,7 | 91 | N03 | 2152 | 0,8 | 93 |
| A07 | 1765 | 0,93 | 91 | R06 | 1842 | 0,6 | 92 | M05 | 1669 | 0,6 | 94 |
| N03 | 1515 | 0,80 | 92 | J01 | 1731 | 0,6 | 92 | N04 | 1414 | 0,5 | 94 |
| V03 | 1229 | 0,65 | 93 | G02 | 1586 | 0,6 | 93 | L02 | 1169 | 0,4 | 95 |
| C05 | 1175 | 0,62 | 93 | R05 | 1477 | 0,5 | 93 | A06 | 1105 | 0,4 | 95 |
| M09 | 1057 | 0,56 | 94 | L02 | 1466 | 0,5 | 94 | A05 | 1033 | 0,4 | 95 |
| L01 | 1007 | 0,53 | 94 | C05 | 1438 | 0,5 | 94 | J01 | 1005 | 0,4 | 96 |
| L02 | 955 | 0,50 | 95 | A06 | 1361 | 0,5 | 95 | R06 | 935 | 0,4 | 96 |
| A06 | 894 | 0,47 | 95 | A07 | 1270 | 0,4 | 95 | L01 | 861 | 0,3 | 96 |
| L03 | 890 | 0,47 | 96 | L01 | 1262 | 0,4 | 96 | N07 | 740 | 0,3 | 97 |
| M03 | 839 | 0,44 | 96 | M05 | 1217 | 0,4 | 96 | A09 | 739 | 0,3 | 97 |
| C04 | 818 | 0,43 | 97 | A09 | 1071 | 0,4 | 97 | M02 | 726 | 0,3 | 97 |
| A03 | 805 | 0,42 | 97 | C04 | 1039 | 0,4 | 97 | C05 | 722 | 0,3 | 98 |

***Appendix 2B:*** *Drug classification corresponding to ATC code*

| (A) Common drugs | |  | | | |  | | | |
| --- | --- | --- | --- | --- | --- | --- | --- | --- | --- |
| ≤ 60 years | | 60 – 70 years | | | | > 70 years | | | |
| ATC code | Drug classification | ATC code | Drug classification | | | ATC code | Drug classification | | |
| C09 | Agents acting on the renin-angiotensinsystem | C09 | Agents acting on the renin-angiotensinsystem | | | C09 | Agents acting on the renin-angiotensinsystem | | |
| H03 | Thyroid therapy | C07 | Beta blocking agents | | | C07 | Beta blocking agents | | |
| C07 | Beta blocking agents | C10 | Lipid modifying agents | | | B01 | Antithrombotic agents | | |
| C10 | Lipid modifying agents | B01 | Antithrombotic agents | | | C10 | Lipid modifying agents | | |
| N02 | Analgesic drugs | H03 | Thyroid therapy | | | C08 | Calcium channel blockers | | |
| N06 | Psychoanaleptics | C08 | Calcium channel blockers | | | H03 | Thyroid therapy | | |
| B01 | Antithrombotic agents | A02 | Drugs for acid related disorders | | | C03 | Diuretic drugs | | |
| M01 | Anti-inflammatory and antirheumatic drugs | N06 | Psychoanaleptics | | | A02 | Drugs for acid related disorders | | |
| A02 | Drugs for acid related disorders | C03 | Diuretic drugs | | | A10 | Drugs used in diabetes | | |
| G02 | Other gynecological drugs | A10 | Drugs used in diabetes | | | G04 | Urological drugs | | |
| C08 | Calcium channel blockers | N02 | Analgesic drugs | | | N06 | Psychoanaleptics | | |
| R03 | Drugs for obstructive airway diseases | M01 | Anti-inflammatory and antirheumatic drugs | | | M01 | Anti-inflammatory and antirheumatic drugs | | |
| C03 | Diuretic drugs | G04 | Urological drugs | | |  |  |  |  |
| A10 | Drugs used in diabetes |  |  |  |  |  |  |  |  |
| N05 | Psycholeptics drugs |  |  |  |  |  |  |  |  |
| M04 | Antigout preparations |  |  |  |  |  |  |  |  |

| (B) Uncommon drugs | |  | |  | |
| --- | --- | --- | --- | --- | --- |
| G04 | Urological drugs | M04 | Antigout preparations | M04 | Antigout preparations |
| R06 | Antihistamines for systemic use | C01 | Cardiac therapy | C01 | Cardiac therapy |
| C01 | Cardiac therapy | R03 | Drugs for obstructive airway disease | N02 | Analgesic drugs |
| J01 | Antibacterial drugs | N05 | Psycholeptics drugs | R03 | Drugs for obstructive airway disease |
| R05 | Cough and cold drugs | C02 | Antihypertensive drugs | C02 | Antihypertensive drugs |
| A05 | Bile and liver therapy | H02 | Corticosteroids systemic | N05 | Psycholeptics drugs |
| H02 | Corticosteroids systemic | A05 | Bile and liver therapy | H02 | Corticosteroids systemic |
| C02 | Antihypertensive drugs | N03 | Antiepileptic drugs | N03 | Antiepileptic drugs |
| A07 | Antidiarrheals, intestinal anti-inflammatory/ anti-infective agents | R06 | Antihistamines for systemic use | M05 | Drugs affecting bone structure and mineralization |
| N03 | Antiepileptic drugs | J01 | Antibacterial drugs | N04 | Antiparkinson drugs |
| V03 | All other therapeutic products | G02 | Other gynecological drugs | L02 | Endocrine therapy |
| C05 | Vasoprotective drugs | R05 | Cough and cold drugs | A06 | Drugs for constipation |
| M09 | Other drugs for disorders of musculo-skeletal system | L02 | Endocrine therapy | A05 | Bile and liver therapy |
| L01 | Antineoplastic drugs | C05 | Vasoprotective drugs | J01 | Antibacterial drugs |
| L02 | Endocrine therapy | A06 | Drugs for constipation | R06 | Antihistamines for systemic use |
| A06 | Drugs for constipation | A07 | Antidiarrheals, intestinal anti-inflammatory/ anti-infective agents | L01 | Antineoplastic drugs |
| L03 | Immunostimulants drugs | L01 | Antineoplastic drugs | N07 | Other nervous system drugs |
| M03 | Muscle relaxants | M05 | Drugs affecting bone structure and mineralization | A09 | Digestives, including enzymes |
| C04 | Peripheral vasodilators | A09 | Digestives, including enzymes | M02 | Topical products for joint and muscular pain |
| A03 | Drugs for functional gastrointestinal disorders | C04 | Peripheral vasodilators | C05 | Vasoprotective drugs |

***Appendix 3****: Most commonly prescribed drugs (ATC Classification) in the EPIC-Heidelberg cohort, by follow-up and sex, including G03*

Pn = participating number of subjects, FUP = follow-up

| **Baseline (Pn=11,928)** | | | **FUP1 (11,011)** | | | **FUP2 (10,309)** | | | **FUP3 (9,854)** | | | **FUP4 (9,468)** | | | **FUP5 (8,755)** | | | **FUP 6 (8,275)** | | |
| --- | --- | --- | --- | --- | --- | --- | --- | --- | --- | --- | --- | --- | --- | --- | --- | --- | --- | --- | --- | --- |
| **ATC** | **N** | **[%]** | **ATC** | **N** | **[%]** | **ATC** | **N** | **[%]** | **ATC** | **N** | **[%]** | **ATC** | **N** | **[%]** | **ATC** | **N** | **[%]** | **ATC** | **N** | **[%]** |
| **MEN** | | |  | | |  | | |  | | |  | | |  | | |  | | |
| **C07** | 1097 | 8.46 | **C07** | 1347 | 9.06 | **C09** | 2147 | 13.58 | **C09** | 2792 | 14.57 | **C09** | 3350 | 15.70 | **C09** | 2671 | 15.02 | **C09** | 3422 | 17.72 |
| **C09** | 907 | 6.99 | **C09** | 1282 | 8.63 | **C07** | 1765 | 11.16 | **C07** | 2109 | 11.01 | **B01** | 2437 | 11.42 | **B01** | 2646 | 14.88 | **B01** | 2540 | 13.16 |
| **N02** | 771 | 5.95 | **B01** | 1076 | 7.24 | **C10** | 1293 | 8.18 | **B01** | 1955 | 10.20 | **C07** | 2377 | 11.14 | **C07** | 2439 | 13.72 | **C07** | 2299 | 11.91 |
| **C10** | 702 | 5.41 | **C10** | 884 | 5.95 | **B01** | 1247 | 7.89 | **C10** | 1759 | 9.18 | **C10** | 2089 | 9.79 | **C10** | 2130 | 11.98 | **C10** | 2113 | 10.94 |
| **C08** | 577 | 4.45 | **G04** | 599 | 4.03 | **G04** | 719 | 4.55 | **C08** | 818 | 4.27 | **G04** | 1037 | 4.86 | **G04** | 1079 | 6.07 | **C08** | 995 | 5.15 |
| **M04** | 529 | 4.08 | **M04** | 580 | 3.90 | **N02** | 678 | 4.29 | **G04** | 762 | 3.98 | **C08** | 942 | 4.42 | **A10** | 1047 | 5.89 | **A10** | 933 | 4.83 |
| **G04** | 507 | 3.91 | **C08** | 573 | 3.86 | **C08** | 672 | 4.25 | **A10** | 750 | 3.91 | **A10** | 908 | 4.26 | **C08** | 815 | 4.58 | **A02** | 855 | 4.43 |
| **A10** | 371 | 2.86 | **N06** | 450 | 3.03 | **M04** | 637 | 4.03 | **M04** | 729 | 3.81 | **M04** | 760 | 3.56 | **A02** | 793 | 4.46 | **G04** | 761 | 3.94 |
| **H03** | 354 | 2.73 | **H03** | 401 | 2.70 | **A10** | 581 | 3.67 | **C03** | 678 | 3.54 | **C03** | 736 | 3.45 | **M04** | 752 | 4.23 | **C03** | 729 | 3.78 |
| **C01** | 345 | 2.66 | **A10** | 389 | 2.62 | **C03** | 506 | 3.20 | **A02** | 569 | 2.97 | **A02** | 714 | 3.35 | **C03** | 566 | 3.18 | **M04** | 680 | 3.52 |
| **Women** | | |  |  |  |  |  |  |  |  |  |  |  |  |  |  |  |  |  |  |
| **G03** | 3703 | 19.08 | **G03** | 3663 | 16.32 | **G03** | 4539 | 20.30 | **G03** | 3005 | 11.83 | **C09** | 2494 | 9.84 | **C07** | 2328 | 13.23 | **C09** | 2710 | 12.93 |
| **H03** | 1640 | 8.45 | **H03** | 1693 | 7.54 | **H03** | 1715 | 7.67 | **C09** | 2017 | 7.94 | **C07** | 2206 | 8.71 | **H03** | 2187 | 12.43 | **C07** | 2163 | 10.32 |
| **C07** | 965 | 4.97 | **C07** | 1166 | 5.20 | **C07** | 1587 | 7.10 | **C07** | 1941 | 7.64 | **H03** | 2032 | 8.02 | **C09** | 2081 | 11.83 | **H03** | 2008 | 9.58 |
| **N06** | 616 | 3.17 | **N06** | 1024 | 4.56 | **C09** | 1516 | 6.78 | **H03** | 1832 | 7.21 | **G03** | 1979 | 7.81 | **B01** | 1577 | 8.96 | **B01** | 1475 | 7.04 |
| **C09** | 570 | 2.94 | **C09** | 841 | 3.75 | **C10** | 873 | 3.90 | **G02** | 1396 | 5.50 | **C10** | 1462 | 5.77 | **C10** | 1483 | 8.43 | **C10** | 1407 | 6.71 |
| **N02** | 492 | 2.54 | **C10** | 568 | 2.53 | **N02** | 803 | 3.59 | **C10** | 1157 | 4.55 | **B01** | 1455 | 5.74 | **A02** | 928 | 5.28 | **G03** | 1037 | 4.95 |
| **C10** | 431 | 2.22 | **B01** | 532 | 2.37 | **N06** | 787 | 3.52 | **B01** | 1099 | 4.33 | **N06** | 967 | 3.82 | **M01** | 790 | 4.49 | **A02** | 994 | 4.74 |
| **C03** | 406 | 2.09 | **N02** | 501 | 2.23 | **G02** | 703 | 3.14 | **N06** | 929 | 3.66 | **G02** | 927 | 3.66 | **G03** | 655 | 3.72 | **A12** | 813 | 3.88 |
| **G02** | 397 | 2.05 | **M01** | 471 | 2.10 | **B01** | 696 | 3.11 | **M01** | 833 | 3.28 | **M01** | 872 | 3.44 | **A10** | 597 | 3.39 | **M01** | 797 | 3.80 |
| **C08** | 390 | 2.01 | **G02** | 421 | 1.88 | **M01** | 608 | 2.72 | **N02** | 701 | 2.76 | **A02** | 812 | 3.21 | **C08** | 584 | 3.32 | **N06** | 787 | 3.75 |
